# Jaw Bone Invasion of Oral Squamous Cell Carcinoma Is Associated with Osteoclast Count and Expression of Its Regulating Proteins in Patients and Organoids

**DOI:** 10.3390/jcm12186035

**Published:** 2023-09-18

**Authors:** Willem W. B. de Kort, Wisse E. Haakma, Robert J. J. van Es, Debby Gawlitta, Else Driehuis, Merel Gansevoort, Stefan M. Willems

**Affiliations:** 1 Department of Pathology, University Medical Center Utrecht, 3584 CX Utrecht, The Netherlands; 2 Department of Oral and Maxillofacial Surgery & Special Dental Care, University Medical Center Utrecht, 3584 CX Utrecht, The Netherlands; r.j.j.vanes@umcutrecht.nl (R.J.J.v.E.); d.gawlitta@umcutrecht.nl (D.G.); 3 Department of Head and Neck Surgical Oncology, Utrecht Cancer Center, University Medical Center Utrecht, 3584 CX Utrecht, The Netherlands; 4 Hubrecht Institute, Developmental Biology & Stem Cell Research, 3584 CT Utrecht, The Netherlands; else.driehuis@xilis.nl; 5 Department of Pathology, University Medical Center Groningen, 9713 GZ Groningen, The Netherlands; s.m.willems@umcg.nl

**Keywords:** head and neck squamous cell carcinoma, bone invasion, mandibular invasion, osteoclasts, RANKL, OPG

## Abstract

Aims: Oral squamous cell carcinoma (OSCC) frequently invades the jaw. The exact mechanism of bone invasion remains unclear. This study investigates (premature) osteoclasts and the expression of its differentiation regulating proteins RANKL, OPG and RANK in patients with OSCC. Methods: Resection specimens from OSCC patients were divided into NI group (No Invasion), E group (Erosion) or I group (bone Invasion). Tissue sections were stained with Cathepsin K (osteoclast-counting), RANKL, OPG and RANK. The staining intensity was scored on different regions of the tumor: front, center, back and normal mucosa. Immunohistochemistry and qPCR for RANKL/OPG/RANK were performed on five head and neck squamous cell carcinoma (HNSCC) organoids. Results: The mean number of osteoclasts (I group) and premature osteoclasts (E group) was significantly higher compared to the NI group (*p* = 0.003, *p* = 0.036). RANKL expression was significantly higher in the tumor front and tumor center compared to normal mucosa (all groups). In the I group, RANKL and RANK expression was significantly higher in the tumor front compared to the tumor back and there was a trend of higher RANKL expression in the tumor front compared to the E group and NI group. qPCR showed a 20–43 times higher RANKL mRNA expression in three out of five tumor organoids compared to a normal squamous cell organoid line. There was no correlation between protein and mRNA expression in the HNSCC organoids. Conclusions: These findings suggest that OSCCs induce bone invasion by stimulating osteoclast activation by regulating the production of RANKL and RANK proteins.

## 1. Introduction

Over 90% of all oral cancers are squamous cell carcinoma (OSCC) [1]. If in contact with bone, OSCC frequently invades the jaw. Cancers of the floor-of-mouth, tongue or retromolar regions invade the jaw in 62%, 42% and 48% of the cases, respectively [2]. In the case of bone invasion, the tumor TNM classification for malignant tumors is staged to the highest T stage of T4 [3]. OSCC is primarily treated with surgery [4]. Patients with bone invasion have worse disease-free survival and overall survival rates [5]. Moreover, the presence of bone invasion is clinically relevant as it often requires a partial resection of the mandible, which has a major impact on quality of life, aesthetics and function [6,7].

Two patterns of mandibular destruction by tumor tissue are recognized: an invasive and an erosive pattern. In the invasive pattern, the tumor breaks through cortical bone and islands of tumor grow into cancellous spaces without an intervening layer of connective tissue. The erosive pattern is characterized by cohesive tumor growth which separates from the bone by an intervening connective tissue layer [8]. Bone invasion starts with bone destruction, which then allows the access of malignant cells. In OSCC, this requires osteoclast differentiation and activation rather than direct growth of tumor cells into the bone [9]. Osteoclasts are multinucleated cells that are differentiated from mononuclear prefusion osteoclasts due to expression of a Receptor Activator of Nuclear factor Kappa-B Ligand (RANKL) [10,11]. RANKL binds its receptor RANK, expressed on hematopoietic osteoclast progenitors and osteoclasts, which induces the differentiation of osteoclasts [9]. Osteoprotegerin (OPG) is the decoy receptor of RANKL. OPG is also known as ‘osteoclastogenesis inhibitory factor’. OPG that binds RANKL prevents RANKL binding RANK thus blocking osteoclast differentiation [12]. RANK and RANKL signaling is also known to contribute to bone invasion or bone metastasis in other types of cancer that are not OSCC [13,14,15,16,17,18,19,20,21].

To further elucidate the mechanisms of bone invasion in OSCC, this study assesses the number of osteoclasts and expression of RANK, RANKL and OPG in a panel of both OSCC patient tissues and head and neck squamous cell carcinoma (HNSCC) organoids, with bone invasion of either the invasive or the erosive pattern. These parameters are compared with a panel of OSCC patients without bone invasion.

## 2. Materials and Methods

### 2.1. Selection of Patients and Clinical Data

This is a retrospective cohort study of patients treated at the University Medical Center Utrecht between January 2016 and December 2018. Inclusion criteria were: (1) patients with OSCC of the upper/lower gum, cheek or mandible, (2) patients treated with primary resection, (3) enough tissue was available for tissue sections and (4) tissue sections were deemed suitable by a dedicated head and neck pathologist (SMW) for assessing the front of the tumor to the bone. As all included tissues were primary resections, the tissues did not receive radiotherapy and/or chemotherapy before surgery. All tissues and data were handled according to the General Data Protection Regulation (GDPR). For all included patients, representative formalin-fixed, paraffin-embedded (FFPE) resection blocks were collected. Then, 4 μm tissue sections of the FFPE blocks were stained with 1:3 diluted hematoxylin for 4 min and with eosin for 1 min (H&E). These stained H&E sections were classified by a head and neck pathologist (SMW) into three categories: No bone Invasion (NI group, tumor did not damage the bone), bone Erosion (E group, tumor eroded the bony cortex but did not grow into the bone) and bone Invasion (I group, tumor did grow into the bone marrow, strands and islands of tumor were identified between the bone trabeculae).

In addition, available tumor tissue of five HNSCCs was cultured as organoids described by Millen et al. [22]. Organoids are three-dimensional tissue cultures derived from stem cells of the tumor. The 5 HNSCC organoid lines originated from the oral cavity (*n* = 1), oropharynx (*n* = 1), nasal cavity (*n* = 1) and the larynx (*n* = 2). The oral cavity organoid was derived from a tumor with bone invasion. One normal mucosa organoid was included as control; this normal mucosa organoid originated from a SCC resection specimen of the oral tongue, from which normal mucosa was used for organoid isolation. The oropharynx organoid was HPV type 16 positive; all the other organoids were HPV negative.

### 2.2. Immunohistochemistry

To recognize and count the number of osteoclasts, tissue sections were stained for Cathepsin K. Alongside, tissue sections and organoids were stained for RANKL, RANK and OPG according to the details displayed in Table 1. All antibody incubation was carried out at room temperature for 1 h. After washing off secondary antibodies, sections were stained with 3,3′-Diaminobenzidine (DAB) for 15 min and counterstained with 1:3 diluted hematoxylin for 30 s.

### 2.3. Counting Osteoclasts and Pre-Osteoclasts

**Figure 1:**
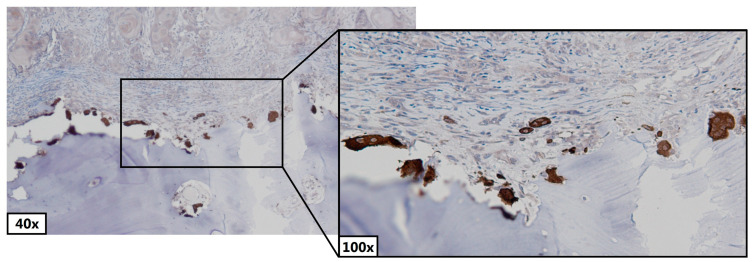
Cathepsin K staining. Multinucleated brown cells depicted are recognized as osteoclasts. Blue depicted area in the bottom half is bone.

The average number of osteoclasts and mononuclear primary osteoclasts (pre-osteoclasts) were counted in the Cathepsin K stained sections by counting them in five, randomly picked, 500 μm wide, areas on the front of the bone close to the tumor. The 500 μm-wide compartments were chosen because this allowed the presence of multiple osteoclasts while retaining the possibility to differentiate osteoclasts and pre-osteoclasts (Figure 1). An osteoclast was recognized as a large multinucleated cell. Mononuclear primary osteoclasts and mature osteoclasts were differentiated. Two trained observers (WdK, MG) and a dedicated head and neck pathologist (SMW) manually counted the number of osteoclasts independently. If there was discordance in osteoclast counting between the three observers, that specific case was assessed together and recounted to reach consensus.

### 2.4. Scoring RANKL, RANK and OPG Intensity Staining

Cytoplasmic RANKL, OPG and RANK expression in tumor (tumor front, tumor center and tumor back) were compared with expression in normal mucosa to see if there were differences in expression between tumor and normal squamous cell mucosa and if there were differences in expression within the tumor towards the bone.

**Figure 2:**
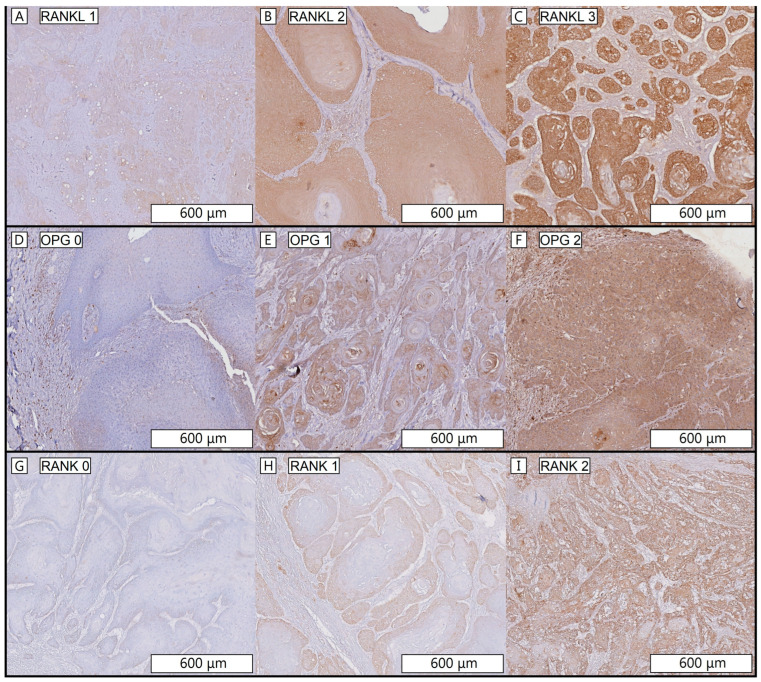
Representative staining intensity score, magnification 6×. RANKL staining intensity (**A**) = 1, (**B**) = 2, (**C**) = 3. OPG staining intensity (**D**) = 0, (**E**) = 1, (**F**) = 2. RANK staining intensity (**G**) = 0, (**H**) = 1, (**I**) = 2. Important note. RANKL is scored on a scale from 0–3 (score 0 for RANKL not shown), OPG and RANK are scored on a scale from 0–2.

Each staining was scored in three tumor areas: tumor front, tumor center and tumor back. “Tumor front” was defined as the area closest to or invading the bone, ”tumor center” as the central part of the tumor and “tumor back” as the area opposite to the tumor front. RANKL staining was scored using a 4-stage intensity scale; 0 negative, 1 light, 2 medium and 3 strong (Figure 2A–C). OPG- and RANK staining both were scored using a 3-stage intensity scale; 0 negative, 1 light, 2 strong (Figure 2D–I). RANKL was scored using a 4-stage intensity scale because the RANKL staining was more discriminative compared to the RANK and OPG stainings. Per staining, every tumor area was scored separately. Three observers scored tissue sections independently (WdK, WH, SMW). In line with the osteoclast counting, if there was discordance between the three observers, expression of that specific case was reassessed together to reach consensus.

### 2.5. Organoid Culture and RNA Isolation

Organoids were cultured as described by Millen et al. [22]. In short, sampled pieces of HNSCC tissue obtained during resections were mechanically disrupted by cutting them into small pieces and were digested enzymatically (0.125% Trypsin, catalog no. T1426, Sigma-Aldrich, Saint Louis, MO, USA). Once tissues were macro and microscopically dissociated, cell suspension was filtered through a 70 uM filter (catalog no. CLS431751-50EA, Corning, Glendale, AZ, USA), resuspended in Cultrex (catalog no. 3533-010-02, Trevigen, Gaithersberg, MD, USA) and plated in droplets of culture medium on 48-well suspension culture plates (catalog no. M9312, Greiner, Kremsmünster, Austria). During the first week of culture, Caspofugin (0.5 mg/mL, Sigma-Aldrich, Saint Louis, MO, USA), an antimycotic, was present and was removed after one week. Medium was changed every two to three days and organoids were passaged between approximately 7 and 14 days after plating, depending on their growth rate. For more details regarding organoid medium see Millen et al. [22].

The Biobank Research Ethics Committee of the University Medical Center Utrecht (TCBio) approved the biobanking protocol: 12-093 HUB-Cancer according to the University Medical Center Utrecht (UMCU) Biobanking Regulation. All donors participating in this study signed informed-consent forms and can withdraw their consent at any time.

RANKL, OPG and RANK immunohistochemistry was performed as described above. For protein scoring the staining intensity of RANKL, OPG and RANK, we used a 3-stage intensity scale; 0 (negative), 1 (light) and 2 (strong). To investigate the mRNA expression levels of RANKL, OPG and RANK in the HNSCC organoids, a quantitative polymerase chain reaction (qPCR) was executed.

Organoids were cultured for twelve days, and RNA was collected after passaging. For RNA collection, organoids were collected from the culture plates by disrupting the basement membrane extract using a p1000 pipette, centrifuged at 300× *g*, 5 min at 4 °C and washed twice in 10 mL medium. RNA was isolated according to protocol using RNeasy Mini Kit of Qiagen. In short, the organoid pellet was lysed in 350 µL RLT buffer, and subsequently incubated 5 min at room temperature. The lysate was stored at −80 °C until further use. On the day of processing, lysate was thawed on ice and 350 µL 70% ethanol was added. Suspension was loaded on columns provided, washed once using 700 µL RW1 buffer and twice using 500 µL RPE buffer. Membranes were spun dry to remove residual buffer, after which the RNA was eluted in 30 uL RNA-free water. RNA concentration was measured using a nanodrop and stored at −80 °C until further use. Only RNA samples with 260/280 ratios ranging from 1.95–2.05 and a concentration > 10 ug/uL were used for subsequent analysis.

### 2.6. cDNA Synthesis and Quantitative PCR

For cDNA synthesis, RNA eluate was thawed on ice, and a volume equal to 500 ng was incubated with 50 μg/mL Oligo (dT) 15 Primer (catalog no. C1101, Promega, Madison, WI, USA) in water for 5 min at 70 °C. To generate cDNA, GoScript Reverse Transcriptase (Promega, catalog no. A5003) was used according to protocol. qPCR reactions were performed in 384-well format using IQ SYBR green (catalog no. 1708880, Bio-Rad, Veenendaal, The Netherlands) in the presence of 0.67 μmol/Lforward and reverse primer (Table S1) and 20 ng of cDNA. For qPCR, samples were incubated for 2 min at 95 °C (initial denaturation) and for 40 cycles at: 15 s at 98 °C (denaturation), 15 s at 58 °C (annealing), and 15 s at 72 °C (extension). Results were calculated by using the delta–delta Ct method, also known as the 2^−∆∆Ct^ method. Expression was expressed relative to expression of the housekeeping gene actin and to RNA isolated from a wildtype tongue epithelium. Melt peak analysis was performed to assure primers used generated only one product. RANKL, OPG and RANK expression on mRNA level was compared to RANKL, OPG and RANK expression on protein level (Figure S2).

### 2.7. Statistics

To assess if there were differences in mean number of (pre)osteoclasts between three patient groups, a one-way ANOVA was used. There were no outliers as assessed by boxplot, data was normally distributed as assessed by Shapiro–Wilk’s test (*p* > 0.05). For osteoclasts there was homogeneity of variances as assessed by Levene’s test (*p* > 0.05) and Tukey–Kramer post hoc testing was used. For premature osteoclasts there was no homogeneity of variances as assessed by Levene’s test (*p* = 0.003), therefore the Welch ANOVA was used with Games–Howell post hoc testing.

As RANKL, OPG and RANK expression is ordinal data, differences in expression were analyzed with non-parametric tests. To compare expression in the tumor front, tumor center, tumor back and normal mucosa the Friedman test was used as expression is compared within the same patient. Pairwise comparisons were performed with a Bonferroni correction for multiple comparisons. To compare expression between patient groups per tumor area, the Kruskal–Wallis H test was used as expression is compared between different patients. Post hoc testing was not performed as the Kruskal–Wallis H tests did not yield significant values.

A *p*-value of <0.05 was interpreted as statistically significant. Statistical analysis was performed with SPSS Statistics (IBM Corp. Released 2017. IBM SPSS Statistics for Windows, Version 26.0. Armonk, NY, USA: IBM Corp).

## 3. Results

### 3.1. Patients

In total 29 patients were included: 7 in the NI group, 12 in the E group and 10 in the I group. Patient characteristics are described in Table 2.

### 3.2. Number of Osteoclasts

**Figure 3:**
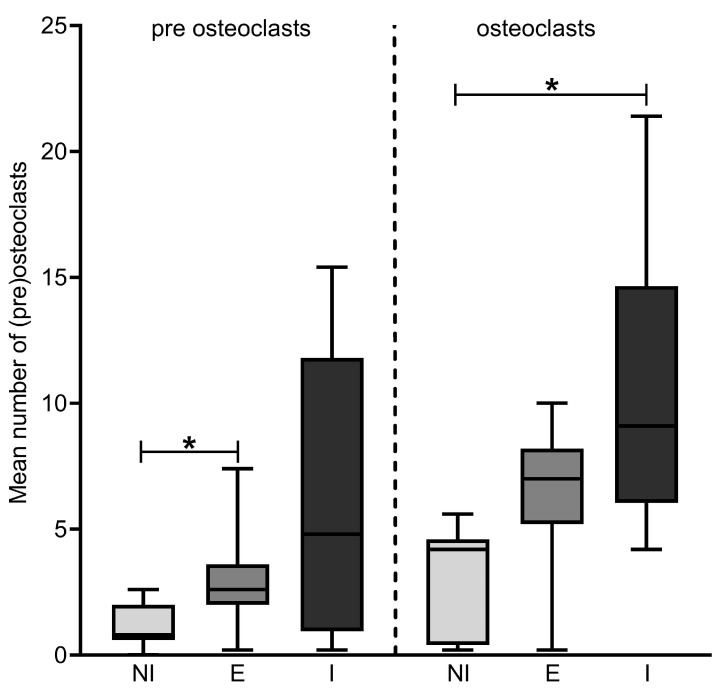
Boxplots mean number of counted osteoclasts with Cathepsin K staining. NI, No Invasion; E, Erosion; I, Invasion. Differences in means between three groups are statistically significant; left Welch’s ANOVA (*p* = 0.015), right one-way ANOVA (*p* = 0.004); * indicates significant difference in group means assessed with post hoc testing.

The mean number of osteoclasts in the Cathepsin K stained sections were 3.09 ± 2.13 (NI group), 6.15 ± 3.13 (E group) and 10.58 ± 5.70 (I group) (Figure 3). These differences in means were statistically significant (*p* = 0.004 one-way ANOVA). Tukey post hoc tests revealed a significant difference in the mean of the I group versus the NI group (7.49, 95%CI 2.42–12.56, *p* = 0.003) and a trend comparing the I group with the E group (4.43, 95%CI −0.13–8.98, *p* = 0.058). The difference in mean of the E group versus the NI group was not statistically significant. The mean number of premature osteoclasts was 1.06 ± 0.91 (NI group), 3.05 ± 2.10 (E group) and 6.03 ± 5.72 (I group) (Figure 3). These differences in means were also statistically significant (*p* = 0.015 Welch’s ANOVA). Games–Howell post hoc tests revealed a significant difference in the mean number of premature osteoclasts of the E group versus the NI group (2.00, 95%CI 0.12–3.87, *p* = 0.036). Other group differences were not statistically significant (I group versus NI group *p* = 0.10, I group versus E group *p* = 0.38).

### 3.3. RANKL, OPG and RANK Stainings

Tissue sections of all 29 patients were stained with RANKL, OPG and RANK. OPG staining failed due to technical issues for 2 patients resulting in an OPG intensity score for 27 patients. Immunohistochemical scores are displayed in Table 3.

### 3.4. Expression in Tumor versus Normal Mucosa

**Figure 4:**
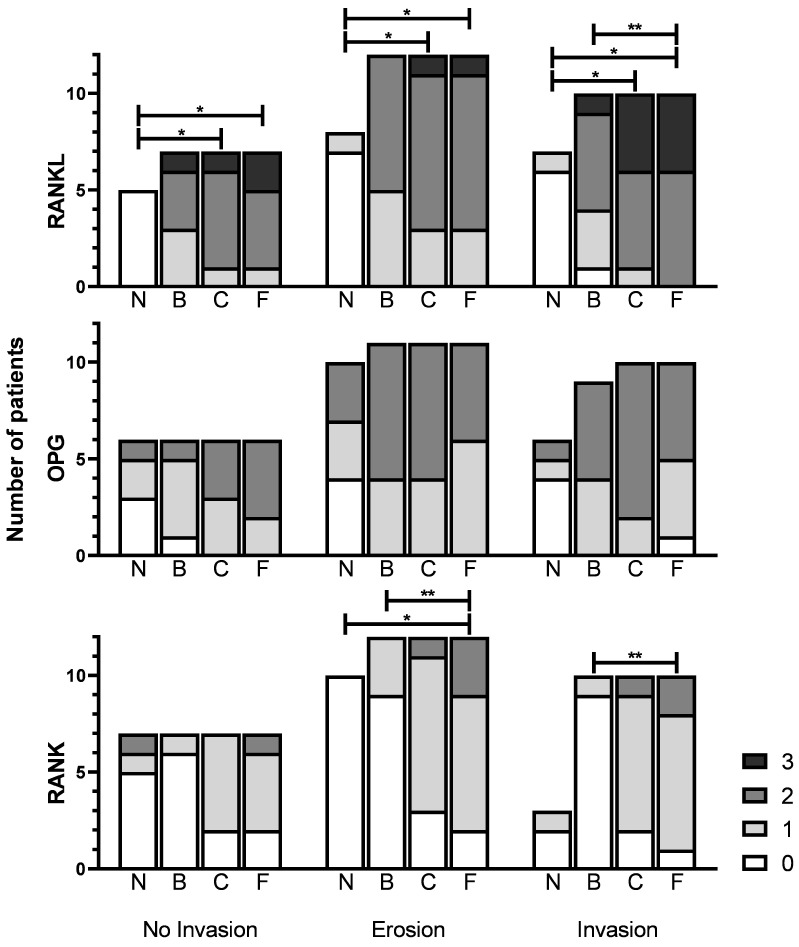
Stacked bar charts of RANKL/OPG/RANK staining intensity score. Legend at the right displays color per staining intensity. X-axis displays three patient groups with subdivision per subsite; N: Normal mucosa, B: Back of tumor, C: Center of tumor, F: Front of tumor. Y-axis displays number of patients. * Displays significant difference in comparison of N, B, C and F in Friedman’s test with multiple comparisons and Bonferroni correction. ** Displays significant difference in comparison of B, C and F in Friedman’s test with multiple comparisons and Bonferroni correction. Important note: RANKL intensity scored 0–3, OPG and RANK intensity scored 0–2.

Cytoplasmic RANKL, OPG and RANK expression in tumors (tumor front, tumor center and tumor back) was compared with expression in normal mucosa (Figure 4, Table S2). RANKL expression, assessed by Friedman’s test, was significantly different in all patient groups comparing normal mucosa, tumor back, tumor center and tumor front; NI group (χ2(3) = 12.60, *p* = 0.006), E group (χ2(3) = 17.855, *p* < 0.001) and I group (χ2(3) = 17.468, *p* = 0.001). Post hoc analysis revealed statistically significant differences in RANKL expression between tumor front and normal mucosa in all three groups; NI group (mean rank 3.50 versus 1.00, *p* = 0.013), E group (mean rank 3.25 versus 1.0, *p* = 0.004) and I group (mean rank 3.57 versus 1.14, *p* = 0.003). Also, post hoc analysis revealed statistically significant differences in RANKL expression between tumor center and normal mucosa in all three groups; NI group (mean rank 3.20 versus 1.00, *p* = 0.042), E group (mean rank 2.94 versus 1.06, *p* = 0.022) and I group (mean rank 3.21 versus 1.14, *p* = 0.016) (Figure 4, Table S2).

OPG expression was assessed by Friedman’s test, and was not significantly different in the NI group and the E group comparing normal mucosa, tumor back, tumor center and tumor front. Although, the I group did significantly differ (χ2(3) = 10.705, *p* = 0.013), post hoc testing revealed no statistically significant differences in OPG expression (Figure 4, Table S2).

RANK expression, assessed by Friedman’s test, was not significantly different in the NI group and I group comparing normal mucosa, tumor back, tumor center and tumor front, but the E group did significantly differ; χ2(3) = 19.857, *p* < 0.001. Post hoc analysis revealed a statistically significant difference in RANK expression between the tumor front (mean rank 3.40) and normal mucosa (mean rank 1.65) (*p* = 0.015) (Figure 4, Table S2).

### 3.5. Expression within the Tumor

RANKL expression, assessed by Friedman’s test, was not significantly different in the NI group and E group comparing tumor front, tumor center and tumor back, but the I group did significantly differ; χ2(2) = 10.571, *p* = 0.005. Post hoc analysis revealed a statistically significant difference in RANKL expression between the tumor front (mean rank 2.40) and tumor back (mean rank 1.30) (*p* = 0.042) (Figure 4, Table S3).

OPG expression, assessed by Friedman’s test, was not significantly different in all patient groups comparing the tumor front, tumor center and tumor back (Figure 4, Table S3).

RANK expression, assessed by Friedman’s test, was significantly different in all patient groups comparing the tumor front, tumor center and tumor back; NI group (χ2(2) = 7.538, *p* = 0.023), E group (χ2(2) = 13.923, *p* = 0.001) and I group (χ2(2) = 14.000, *p* = 0.001). Post hoc analysis revealed a statistically significant difference in RANK expression between the tumor front (mean rank 2.46) and tumor back (mean rank 1.38) (*p* = 0.024) in the E group and a statistically significant difference in RANK expression between the tumor front (mean rank 2.50) and tumor back (1.25) (*p* = 0.016) in the I group. For the NI group, post hoc testing revealed no statistically significant differences in RANK expression (Figure 4, Table S3).

### 3.6. Expression between Invasion Categories

RANKL, OPG and RANK expression were not statistically significantly different when comparing the patient groups; no invasion, erosion and invasion, per tumor side (tumor front, tumor center and tumor back) (Kruskal–Wallis H test). However, there was a trend of higher RANKL expression in the tumor front in patients with bone invasion compared to patients with erosion and without invasion (*p* = 0.10) (Figure 4 and Figure S3, Table S4).

### 3.7. Organoid Staining and qPCR

**Figure 5:**
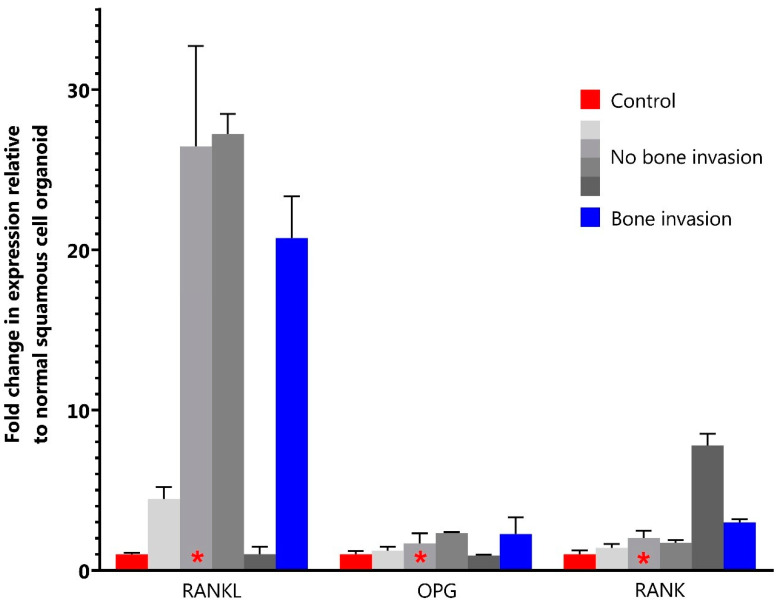
Results of quantitative PCR. * indicates HPV positive organoid line. Red: indicates organoid line of wildtype tongue epithelium used as control; Blue: indicates organoid line with bone invasion; Gray: indicates 4 organoid lines without bone invasion.

Immunohistochemically, all organoids scored medium intensity for RANKL, OPG and RANK. There was no difference in RANKL, OPG and RANK expression between organoids with and without bone invasion. Quantitative PCR showed a 20–43 times higher expression of RANKL in three out of five HNSCC organoids compared to expression in the normal mucosa organoid (Figure 5 and Figure S1). For OPG and RANK, qPCR did not show higher mRNA expression compared to expression in a normal mucosa organoid. There was no correlation between RANKL/OPG/RANK protein and RANKL/OPG/RANK mRNA expression in the HNSCC organoids (Figure S2).

## 4. Discussion

The presence of bone invasion in OSCC has clinical relevance because it may influence the extent of mandibular or maxillary resection. In OSCC it is known that osteoclast differentiation and activation contributes significantly to bone invasion rather than direct growth of tumors in bone [9].

In this study we aimed to unravel some of the underlying mechanisms of bone invasion. We showed that bone invasion is associated with a higher osteoclast count compared to patients without bone invasion, and found a strong trend of higher osteoclast count comparing patients with bone invasion and erosion (*p* = 0.06). The absolute number of premature osteoclasts was significantly higher in patients with bony erosion compared to patients without invasion. A possible explanation for these findings could be that bone-erosive tumors recruit premature osteoclasts which fuse and become multinucleated active osteoclasts resulting in bone destruction whereby the tumor progresses to a bone-invading tumor. Apart from our study, two other studies report a ‘’mixed’’ pattern where progression is possible from the erosive to the invasive pattern [2,23].

In contrast, Carter et al. showed an accumulation of osteoclasts in the erosive phase, leading to bone resorption ahead of the tumor, but a decrease in osteoclast amount in the invasive phase where the tumor destructs the bone itself [24]. A possible explanation for these differences in results is the location where we measured the number of osteoclasts. We measured the number of osteoclasts at the location where the tumor enters the bone. The front of an invasive tumor could already have passed this ‘entering’ area and this front of the tumor destructs the bone itself so the osteoclast count could be low. At the ‘entering’ area we measured a high osteoclast count, as the tumor accumulates osteoclasts for entering the bone. Apart from head and neck squamous cell carcinoma, two studies describe the ability of human breast cancer, prostate cancer and bone metastasis to induce osteolysis by stimulating osteoclasts [25,26].

Apart from the absolute number of osteoclasts, we investigated RANKL, OPG and RANK that regulate osteoclast differentiation. This study showed a significantly higher RANKL expression in tumors compared to normal mucosa, indicating that tumor cells can express RANKL. These findings are in line with the study of Chuang et al. which also found increased immunohistochemical RANKL expression in tumors compared to normal mucosa [27]. This indication is confirmed in our q-PCR, where HNSCC organoids from different subsites, and HPV status, express higher RANKL compared to a non-malignant squamous cell organoid line.

Additionally, we showed there was significantly higher RANKL expression in the tumor front versus the tumor back in the invasion group. For the other groups, a visible trend is present of increasing RANKL staining towards the tumor front. When comparing patient groups, a trend was visible of high RANKL expression in the tumor front in the I group compared to the E group and NI group (*p* = 0.10). Cui et al. also found higher RANKL expression in patients with bone invasion compared to patients without bone invasion [28]. A possible hypothesis of this could be that the tumor encounters bone and increases RANKL expression to induce osteoclastogenesis for osteolysis and hereby becomes an invasive tumor. A higher RANK expression in the tumor front compared to the tumor back supports this hypothesis. In addition to this, Elmusrati et al. 2017 describe that RANKL is expressed in tumors proximal to bone and plays an important role in bone invasion in OSCC [29]. As previously described, RANKL expression in the tumor front was highest in patients with bone invasion. However, these differences were not statistically significant which may have been caused by the limited sample size of our study. Nevertheless, there was a visible trend.

Apart from OSCC, RANK and RANKL signaling is also known to contribute to bone invasion or bone metastasis in myeloma, breast, hepatocellular, lung and prostate cancer [13,14,15,16,17,18,19,20,21]. It is known that osteoclastogenesis can be induced by squamous cell carcinoma, myeloma and promyelocytic leukemia cells expressing RANKL [10,11]. We found no significant differences in OPG expression related to bone invasion. However, in one study a higher OPG expression correlated with infiltrative bone invasion [30].

It is interesting to consider RANKL as a potential ‘bone-invasion-inhibiting-target’, as OSCC is capable of producing RANKL. Denosumab is a monoclonal RANKL inhibitor and is clinically administered in patients with metastatic bone lesions [31,32,33,34]. In patients that received Denosumab, bone metastasis and/or skeletal-related events occurred significantly later compared to patients receiving zoledronic acid. Despite bone invasion in OSCC affecting a different anatomical subsite compared to patients with osteolytic metastasis, the mechanism of bone invasion is comparable. Good et al. investigated RANKL expression in several osteolytic bone tumors and metastasis and concluded that tumor tissue is capable of expressing RANKL [35], which is in line with our study. In palliative treatment of a local recurrence or bone metastasis, suppressing bone invasion by RANKL inhibition could be interesting as bone-invading tumors prefer nutrient-rich bone, which may lead to more tumor growth [9]. Inhibiting bone invasion could constrain tumor growth. Implementing Denosumab as a treatment in primary OSCC is difficult as in some cases the tumor has already invaded the jawbone at the time of diagnosis. However, understanding the mechanism of bone invasion, which this study explored, is crucial to investigate new options for implementing Denosumab. The role of Denosumab in head and neck cancer has yet to be elucidated as, to our knowledge, no studies have investigated this issue.

To investigate whether the protein expression of RANKL, OPG and RANK correlated with mRNA expression, we compared these two groups. There appeared no correlation between protein and mRNA expression in the HNSCC organoids, meaning that RANKL, OPG and RANK could be regulated post-transcriptionally (Figure S2). However, with only five included organoids it is hard to draw such firm conclusions. The limited sample size affects the power of the study. Moreover, the five organoid lines originated from different locations in the head and neck area and one organoid was HPV positive. On the other hand, they are all HNSCC organoids and therefore basically comparable.

This study has several limitations. First, the limited sample size of the tissue slides hampers drawing firm conclusions, which is due to the fact that the number of cases encountered per year is limited. Secondly, the sample size of the organoids is small as described above, and thirdly the way of analyzing the tissue slides with many parameters could introduce statistically significant findings by chance that are not clinically relevant. In conclusion, this study shows that bone-invasive and erosive OSCCs have more osteoclasts (invasion) and premature osteoclasts (erosion) at the tumor front compared to OSCCs without invasion and that OSCCs can express RANKL regardless of bone invasion. Also, in patients with bone invasion, RANKL and RANK expression in the tumor front is significantly higher compared to the tumor back and there is a trend of higher RANKL expression in the tumor front compared to patients with erosion and without invasion. Apart from this, this study describes the capability of HNSCC organoids to express RANKL on protein and mRNA levels. These findings suggest that OSCCs induce bone invasion by stimulating osteoclast activation by regulating the production of RANKL and RANK proteins.

## Figures and Tables

**Table 1:** Antibody stains and dilutions.

Antibody	Manufacturer	Ordering Number	Antigen Retrieval	Primary Antibody Dilution	Secondary Antibody
Cathepsin K	Abcam, Cambridge, UK	ab19027	pH6 Citrate	1:500	Goat anti-rabbit HRP
RANKL	Abcam, Cambridge, UK	ab13918	pH6 citrate	1:100	Goat anti-rabbit HRP
RANK	Abcam, Cambridge, UK	ab9957	pH9 EDTA	1:100	Goat anti-mouse HRP
OPG	Abcam, Cambridge, UK	ab183910	pH9 EDTA	1:200	Goat anti-rabbit HRP

**Table 2:** Patient characteristics; * Primary intraosseous carcinoma (PIOC); Male (M), Female (F).

	No Invasion (*n* = 7)	Erosion (*n* = 12)	Invasion (*n* = 10)
Age (Median (range)	67 (43–88)	72 (60–79)	63 (40–79)
Gender (M:F)	1:6	8:4	5:5
Tumor site			
- Lower gum	4	9	6
- Upper gum	2	3	3
- Cheek	1		
- Mandible *			1
Growth Pattern			
- Cohesive	5	5	4
- Non-cohesive	2	7	6
Peri-neural invasion			
- No	7	3	4
- Yes	0	9	6
Angioinvasion			
- No	7	12	9
- Yes	0	0	1

**Table 3:** Immunohistochemical scores RANKL/RANK/OPG per patient group. Each number indicates number of patients with the corresponding expression score.

	**No Invasion (*n* = 7)**				**Erosion (*n* = 12)**				**Invasion (*n* = 10)**			
**RANKL Score**	**0**	**1**	**2**	**3**	**0**	**1**	**2**	**3**	**0**	**1**	**2**	**3**
Normal mucosa *	5*	0	0	0	7	1	0	0	6	1	0	0
Back of tumor	0	3	3	1	0	5	7	0	1	3	5	1
Center of tumor	0	1	5	1	0	3	8	1	0	1	5	4
Front of tumor	0	1	4	2	0	3	8	1	0	0	6	4
	**No Invasion (*n* = 6)**				**Erosion (*n* = 11)**				**Invasion (*n* = 10)**			
**OPG Score**	**0**	**1**	**2**		**0**	**1**	**2**		**0**	**1**	**2**	
Normal mucosa **	3	2	1		4	3	3		4	1	1	
Back of tumor ***	1	4	2		0	4	7		0	4	5	
Center of tumor	0	3	3		0	4	7		0	2	8	
Front of tumor	0	2	4		0	6	5		1	4	5	
	**No Invasion (*n* = 7)**				**Erosion (*n* = 12)**				**Invasion (*n* = 10)**			
**RANK Score**	**0**	**1**	**2**		**0**	**1**	**2**		**0**	**1**	**2**	
Normal mucosa ****	5	1	1		10	0	0		2	1	0	
Back of tumor	6	1	0		9	3	0		9	1	0	
Center of tumor	2	5	0		3	8	1		2	7	1	
Front of tumor	2	4	1		2	7	3		1	7	2	

* Expression of RANKL in normal mucosa could not be assessed for 2 patients without invasion, 4 patients with erosion and 3 patients with invasion. ** Expression of OPG in normal mucosa could not be assessed for 1 patient with erosion and 4 patients with invasion. *** Expression of OPG in the back of the tumor could not be assessed for 1 patient with invasion. **** Expression of RANK in normal mucosa could not be assessed for 2 patients with erosion and 7 patients with invasion.

## Data Availability

The datasets generated during and/or analyzed during the current study are available from the corresponding author on reasonable request.

## Supplementary Materials

The following supporting information can be downloaded at: https://www.mdpi.com/article/10.3390/jcm12186035/s1, Figure S1: Results of 2nd quantitative PCR; Figure S2: Protein versus mRNA expression; Figure S3: Differences in expression between patient groups per tumor side. Stacked bar charts of RANKL/OPG/RANK staining intensity score; Table S1: Primers for qPCR; Table S2: Statistical testing of RANKL/OPG/RANK expression comparing tumor front, tumor center and tumor backside with expression in normal mucosa; Table S3: Statistical testing of RANKL/OPG/RANK expression comparing tumor front, tumor center and tumor backside.
