# Supplementary material for: Jaw Bone Invasion of Oral Squamous Cell Carcinoma Is Associated with Osteoclast Count and Expression of Its Regulating Proteins in Patients and Organoids
Source: J Clin Med. 2023 Sep 18;12(18):6035. doi: 10.3390/jcm12186035 (PMC10531999; doi:10.3390/jcm12186035)
Supplement: Supplementary file 1 [file jcm-12-06035-s001.zip › jcm-2595707-supplementary.pdf]

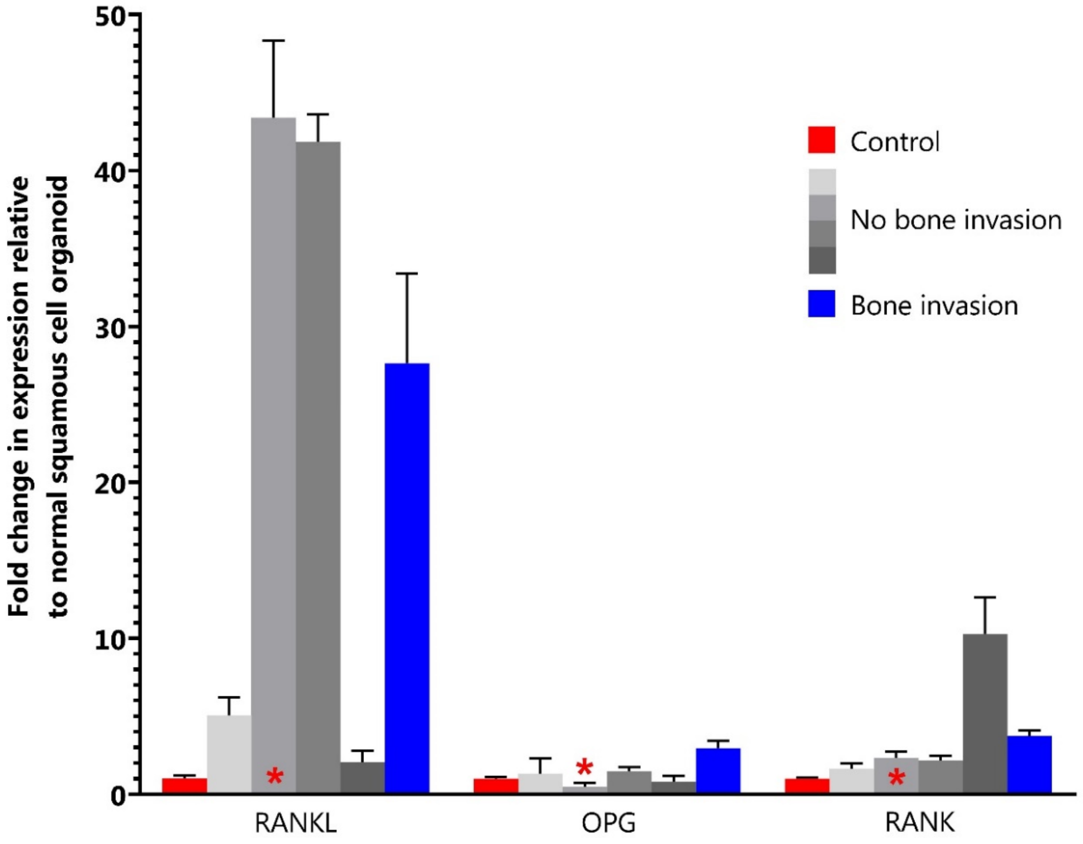

Figure S1. Results of 2nd quantitative PCR, *indicates HPV positive organoid line. Red: indicates organoid line of wildtype tongue epithelium used as control; Blue: indicates organoid line with bone invasion; Grey: indicates 4 organoid lines without bone invasion.

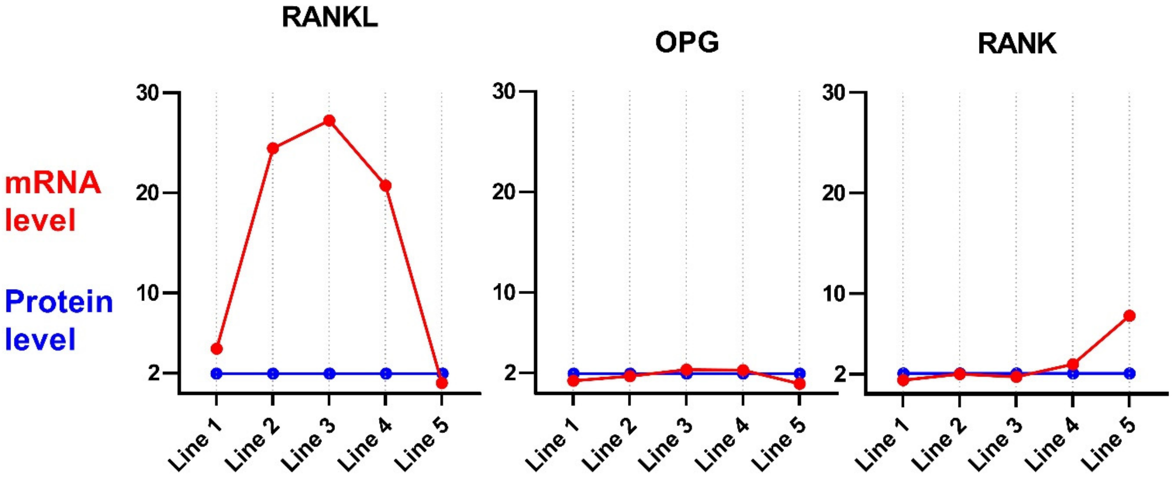

Figure S2. Protein versus mRNA expression, Immunohistochemical protein expression of RANKL, OPG and RANK was compared with mRNA expression assessed with qPCR.

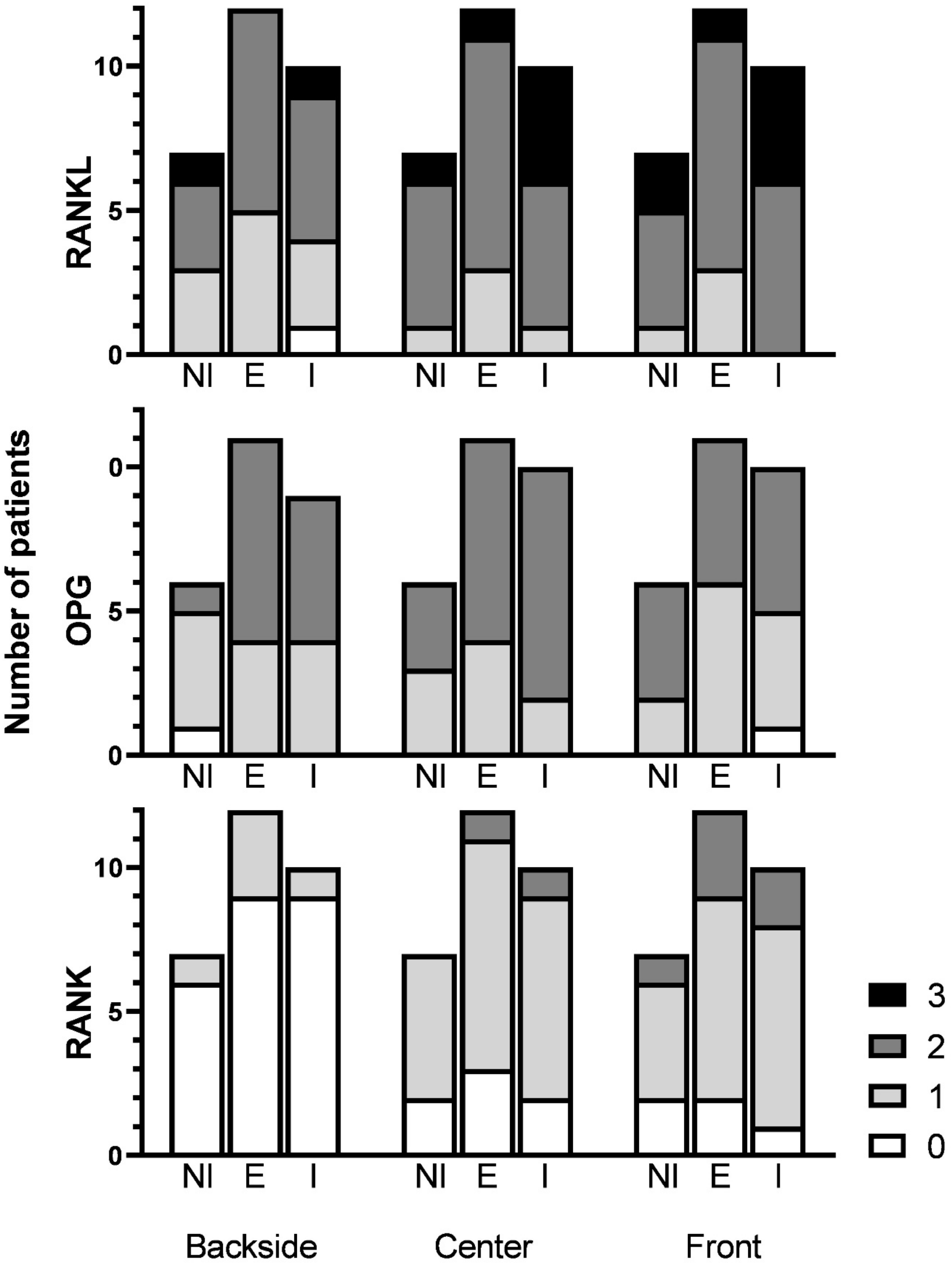

Figure S3. Differences in expression between patient groups per tumor side. Stacked bar charts of RANKL/OPG/RANK staining intensity score. Legend at the right displays color per staining intensity. X-axis displays three tumor sides with subdivision per patient group; NI: No Invasion, E: Erosion, I: Invasion. Y-axis displays number of patients. Groups were analyzed using Kruskal-Wallis H test, differences between groups were not statistically significant. Important note: RANKL intensity scored 0-3, OPG and RANK intensity scored 0-2.

Table S1. Primers for qPCR

primer name		primer sequence	length of product
Human RANKL(TNFSF11)	Forward 1	CAACATATCGTTGGATCACAGCA	161
Human RANKL(TNFSF11)	Reverse 1	GACAGACTCACTTTATGGGAACC	
Human RANKL(TNFSF11)	Forward 2	CCCATAAAGTGAGTCTGTCC	256
Human RANKL(TNFSF11)	Reverse 2	CAATACTTGGTGCTTCCTCC	
Human OPG(TNFRSF11B)	Forward 1	CACAAATTGCAGTGTCTTTGGTC	216
Human OPG(TNFRSF11B)	Reverse 1	TCTGCGTTTACTTTGGTGCCA	
Human OPG(TNFRSF11B)	Forward 2	GAAGGGCGCTACCTTGAGAT	102
Human OPG(TNFRSF11B)	Reverse 2	GCAAACTGTATTTCGCTCTGG	
Human RANK(TNFRSF11A)	Forward 1	TCCTCCACGGACAAATGCAG	92
Human RANK(TNFRSF11A)	Reverse 1	CAAACCGCATCGGATTTCTCT	
Human RANK(TNFRSF11A)	Forward 2	CACCAAATGAACCCCATGTTTAC	182
Human RANK(TNFRSF11A)	Reverse 2	GGACTCCTTATCTCCACTTAGGC	

Table S2: Differences in expression in tumor compared to expression in normal mucosa

	**Friedman test**		**Multiple comparison with Bonferroni correction**		
	Test statistic	p-value test statistic	Tumor front versus Normal mucosa	Tumor center versus Normal mucosa	Tumor backside versus Normal mucosa
**RANKL**					
No invasion	$\chi2(3) = 12.60$	0.006	**0.013**	**0.042**	0.668
Erosion	$\chi2(3) = 17.855$	<0.001	**0.004**	**0.022**	0.054
Invasion	$\chi2(3) = 17.468$	0.001	**0.003**	**0.016**	1.000
**OPG**					
No invasion	$\chi2(3) = 6.480$	0.09	*	*	*
Erosion	$\chi2(3) = 3.766$	0.288	*	*	*
Invasions	$\chi2(3) = 10.705$	0.013	0.877	0.061	0.152
**RANK**					
No invasion	$\chi2(3) = 7.235$	0.065	*	*	*
Erosion	$\chi2(3) = 19.857$	<0.001	**0.015**	0.116	1.000
Invasion	$\chi2(3) = 7.174$	0.067	*	*	*

Table S2: Statistical testing of RANKL/OPG/RANK expression comparing tumor front, tumor center and tumor backside with expression in normal mucosa. As the expression score is ordinal data and expression is compared within a patient, the non-parametric Friedman's test was used. If the Friedman's test was statistically significant, multiple comparison with Bonferroni correction was executed. A p-value of ≤0.05 was interpreted as statistical significant and is displayed in bold.

Table S3: Differences in expression within tumor

	**Friedman test**		**Multiple comparison with Bonferroni correction**		
	Test statistic	p-value test statistic	Tumor front versus Tumor backside	Tumor center versus Tumor backside	Tumor front versus Tumor center
**RANKL**					
No invasion	$\chi2(2) = 2.333$	0.311	*	*	*
Erosion	$\chi2(2) = 2.571$	0.276	*	*	*
Invasion	$\chi2(2) = 10.571$	0.005	**0.042**	0.076	1.000
**OPG**					
No invasion	$\chi2(2) = 2.947$	0.229	*	*	*
Erosion	$\chi2(2) = 4.000$	0.135	*	*	*
Invasions	$\chi2(2) = 2.273$	0.321	*	*	*
**RANK**					
No invasion	$\chi2(2) = 7.538$	0.023	0.247	0.425	1.000
Erosion	$\chi2(2) = 13.923$	0.001	**0.024**	0.157	1.000
Invasion	$\chi2(2) = 14.000$	0.001	**0.016**	0.076	1.000

Table S3: Statistical testing of RANKL/OPG/RANK expression comparing tumor front, tumor center and tumor backside. As the expression score is ordinal data and expression is compared within a patient, the non-parametric Friedman's test was used. If the Friedman's test was statistically significant, multiple comparison with Bonferroni correction was executed. A p-value of ≤0.05 was interpreted as statistical significant and is displayed in bold.

Table S4: Differences in expression between patient groups per tumor side

	**Mean Rank**			**Kruskal Wallis Test**	
	No invasion	Erosion	Invasion	Test statistic	p-value
**RANKL**	N=7	N=12	N=10		
Tumor-front	15.50	11.79	18.50	4.587	0.101
Tumor-center	14.57	12.63	18.15	3.102	0.212
Tumor-Backside	15.64	14.58	15.05	0.085	0.958
**OPG**	N=6	N=11	N=10		
Tumor-front	16.17	13.41	13.35	0.743	0.690
Tumor-center	11.75	13.59	15.80	1.536	0.464
Tumor-backside	8.50	15.45	14.44	4.397	0.111
**RANK**	N=7	N=12	N=10		
Tumor-front	12.93	15.58	15.75	0.733	0.693
Tumor-center	13.64	15.04	15.90	0.440	0.802
Tumor-backside	14.57	16.13	13.95	0.885	0.642

Table S4: Statistical testing of RANKL/OPG/RANK expression comparing patient groups; no invasion, erosion and invasion per tumor side (tumor front, tumor center and tumor backside). As the expression score is ordinal data and expression is compared from different patients, the non-parametric Kruskal-Wallis H test was used. As none of these tests were statistically significant, post-hoc testing was not executed. A p-value of ≤0.05 was interpreted as statistical significant and is displayed in bold.
